# Uncommon external abdominal oblique muscle strain in a professional soccer player: a case report

**DOI:** 10.1186/1756-0500-7-684

**Published:** 2014-10-01

**Authors:** Marc Dauty, Pierre Menu, Charles Dubois

**Affiliations:** CHU Nantes, Service de MPR Locomoteur, Hôpital Saint Jacques, 85 rue Saint Jacques, Cedex 01, 44 035 Nantes, France

**Keywords:** Soccer injury, External oblique muscle, Platelet-rich plasma

## Abstract

**Background:**

This is the first report of external abdominal oblique muscle injury occurring in a professional soccer player.

**Case presentation:**

A 28-year-old Caucasian professional soccer player presented after experiencing a popping sensation associated with strong parietal pain localized between the left 11th and 12th ribs. Ultrasound examination revealed a collection of fluid under the 11th rib, suggesting injury of the left external oblique muscle. Platelet-rich plasma treatment was administered and the soccer player returned to competition on the 21st day after treatment.

**Conclusion:**

This rare injury results from a sudden intrinsic eccentric contraction of the internal oblique muscle while in a stretched position. Ultrasound can help to confirm the diagnosis and to monitor clinical follow-up. Platelet-rich plasma treatment could aid recovery in high-level athletes.

## Background

Lateral trunk muscle injuries are often linked to participation in sports that require trunk rotation, such as tennis or handball, but rarely occur in soccer players [1–3]. The internal oblique muscle is more often injured than the external oblique muscle; the two muscles together form a complex that controls trunk rotation and lateral trunk flexion. No case of external oblique muscle injury has been reported in association with soccer practice.

## Case presentation

A 28-year-old Caucasian professional soccer player presented with left parietal pain 4 hours after soccer training and 24 hours after a session of trunk strengthening, without any prior history of similar signs. The patient’s pain worsened with sneezing, and disappeared during abdominal contraction and intense respiratory movement. Palpation revealed intercostal pain between the left 11th and 12th ribs. Abdominal muscle contraction and isometric exercises were painless.

Thoracic spine examination, costal and abdominal visceral palpation, and pulmonary auscultation were within normal limits. The patient was able to continue practicing, with only intense running causing extreme pain. Analgesic treatment did not relieve this mechanical pain, which was absent in activities of daily living. Four days after the onset of pain, a competition was scheduled and the player was feeling ready to participate.

**Figure 1:**
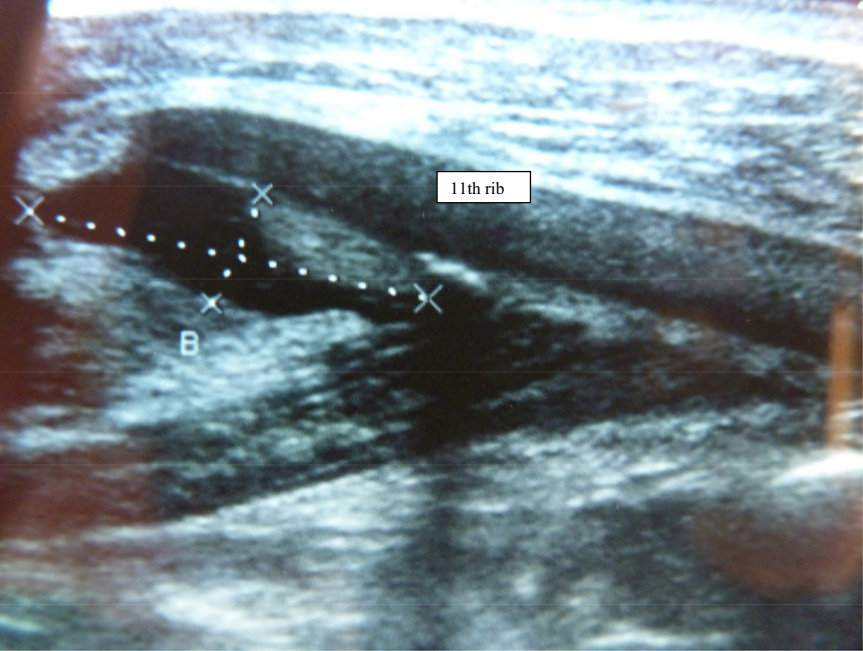
**A 4-cm injury of the left external oblique muscle.**

Fifteen minutes into the match, the patient experienced violent impact to his right shoulder, with extension of the ribcage on the left side. He experienced a popping sensation associated with strong left parietal pain localized at the level of his previous complaint and had to leave the match. The patient’s abdomen was sensitive during trunk rotation. Sitting was also very difficult, while walking was painless. Treatment with a level 1 analgesic (paracetamol) and local icing were prescribed. A bruise appeared 12 hours after injury, and a 3-cm deep tumefaction indicated a hematoma. Ultrasound highlighted fluid collection under the 11th rib, suggesting a 4-cm injury of the left external oblique muscle (Figure 1). There were no signs of rib fracture on X-rays.

**Figure 2:**
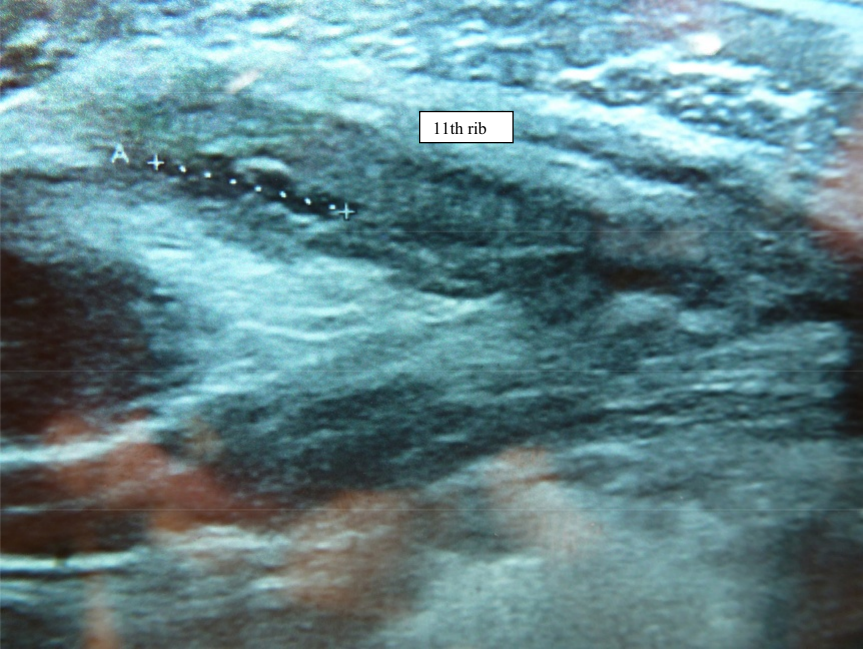
**Progressive resolution of the external oblique muscle injury (day 15).**

At the completion of icing therapy, pain was present only during spinal extension and during contraction of the trunk muscles. Treatment with platelet-rich plasma (Proteal®, Barcelona, Spain) was prescribed on the fourth day after injury, with the patient’s consent. After administration of a local anesthetic (1% lidocaine chlorhydrate), 4 mL of platelet-rich plasma was injected in the region of the injury. The patient was advised not to participate in sports for 3 days. The patient was able to bike on the 4th day after injection, to run on the 5th day, to play soccer on the 15th day, and to compete on the 21st day. Trunk strengthening exercises were resumed on day 15. Ultrasound examination on day 15 revealed progressive healing of the injury (Figure 2). The patient returned to professional soccer practice with no recurrence of his symptoms during 12-month follow-up.

## Discussion

The literature offers accurate analyses of external oblique muscle injury. Three cases associated with cricket and several cases in baseball players have been reported [2, 4]. The acute pain experienced with this type of injury makes continuing practice impossible. In these cases, the test of resisted side bending is always painful. Cricket players are required to rest from their sport for 28–70 days. The injury most commonly occurs at the 10th rib, but can extend from the 9th to the 11th ribs, based on the topography of the muscle insertions [4]. Nuclear magnetic resonance imaging is generally performed for professional athletes. However, this case and others have confirmed that ultrasound is effective in diagnosing abdominal muscle injuries [3, 5]. Ultrasound follow-up has not been described, and healing has been assessed by progressive decrease in the size of the injury. Thus, the treatment was based on functional improvement, because correlation between clinical signs and ultrasound findings is lacking. In our patient, rib mobility and muscle strength both recovered in conjunction with a progressive reduction in pain over a 15-day period. At 15 days, the patient restarted muscular strengthening exercises to stabilize the trunk.

Initial treatment with rest, ice, and physiotherapy is usually recommended for oblique muscle injury. Humphries et al. also recommended anti-inflammatory treatment [4]. Corticosteroid injections are sometimes administered, but there is no evidence of their efficacy for this condition [3, 5]. We chose to inject platelet-rich plasma into the site of injury, under local anesthetic and with the patient’s consent, to improve pain relief and to bring growth factors to the site of the injury [6]. However, evidence is needed to show that this treatment hastens healing. A shorter, 21-day rest period is possible in soccer players, because unlike cricket, soccer does not require the use of the arms, although trunk stabilization is necessary. This kind of muscle injury can recur over time, although our patient has not experienced recurrence.

## Conclusion

This clinical report describes a rare case of external abdominal oblique muscle injury in a professional soccer player. The diagnosis was confirmed by ultrasound. Platelet-rich plasma was easily injected under ultrasound guidance to promote faster healing.

## Consent

Written informed consent was obtained from the patient for publication of this Case Report and any accompanying images. A copy of the written consent is available for review by the Editor-in-Chief of this journal.
